# Cysteamine re-establishes the clearance of *Pseudomonas aeruginosa* by macrophages bearing the cystic fibrosis-relevant F508del-CFTR mutation

**DOI:** 10.1038/cddis.2016.476

**Published:** 2017-01-12

**Authors:** Eleonora Ferrari, Romina Monzani, Valeria R Villella, Speranza Esposito, Francesca Saluzzo, Federica Rossin, Manuela D'Eletto, Antonella Tosco, Fabiola De Gregorio, Valentina Izzo, Maria C Maiuri, Guido Kroemer, Valeria Raia, Luigi Maiuri

**Affiliations:** 1 Division of Genetics and Cell Biology, San Raffaele Scientific Institute, European Institute for Research in Cystic Fibrosis, Milan 20132, Italy; 2 Department of Biology, University of Rome 'Tor Vergata', Rome, Italy; 3 Regional Cystic Fibrosis Center, Pediatric Unit, Department of Translational Medical Sciences, FedericoII University Naples 80131, Italy; 4 Equipe11 labellisée Ligue Nationale contrele Cancer, Centre de Recherche des Cordeliers, Paris, France; 5 INSERM U1138, Centre de Recherche des Cordeliers, Paris, France; 6 Université Paris Descartes, Paris, France; 7 Metabolomics and Cell Biology Platforms, Institut Gustave Roussy, Villejuif, France; 8 Pôlede Biologie, Hôpital Européen Georges Pompidou, AP-HP, Paris, Franceand; 9 Karolinska Institute, Department of Women's and Children's Health, Karolinska University Hospital, Stockholm 17176, Sweden; 10 SCDU of Pediatrics, Department of Health Sciences, University of Piemonte Orientale, Novara 28100, Italy

## Abstract

Cystic fibrosis (CF), the most common lethal monogenic disease in Caucasians, is characterized by recurrent bacterial infections and colonization, mainly by *Pseudomonas aeruginosa*, resulting in unresolved airway inflammation. CF is caused by mutations in the gene coding for the cystic fibrosis transmembrane conductance regulator (CFTR) protein, which functions as a chloride channel in epithelial cells, macrophages, and other cell types. Impaired bacterial handling by macrophages is a feature of CF airways, although it is still debated how defective CFTR impairs bacterial killing. Recent evidence indicates that a defective autophagy in CF macrophages leads to alterations of bacterial clearance upon infection. Here we use bone marrow-derived macrophages from transgenic mice to provide the genetic proof that defective CFTR compromises both uptake and clearance of internalized *Pseudomonas aeruginosa*. We demonstrate that the proteostasis regulator cysteamine, which rescues the function of the most common F508del-CFTR mutant and hence reduces lung inflammation in CF patients, can also repair the defects of CF macrophages, thus restoring both bacterial internalization and clearance through a process that involves upregulation of the pro-autophagic protein Beclin 1 and re-establishment of the autophagic pathway. Altogether these results indicate that cysteamine restores the function of several distinct cell types, including that of macrophages, which might contribute to its beneficial effects on CF.

Cystic fibrosis (CF), the most common lethal monogenic disease in Caucasians, is caused by mutations in the gene coding for cystic fibrosis transmembrane conductance regulator (CFTR), a 1480 amino-acid protein that functions as a chloride channel at the plasma membrane.^1^ Approximately 2000 mutations, most of which are disease relevant, have been identified in the CFTR gene and then categorized in six different classes according to their functional impact.^2^ CF is a systemic disease, characterized by highly heterogeneous manifestations in distinct organs,^1, 3, 4^ comprising insufficiency of the exocrine pancreas, increased electrolytes in sweat, male infertility, and, most prevalent, a chronic progressive lung disease resulting from decreased mucociliary clearance with accumulation of thick, sticky mucus, chronic inflammation, and persistent and untreatable bacterial infections and colonization, mainly by *Pseudomonas aeruginosa* (*P. aeruginosa*), *Staphilococcus aureus* and *Burkholderia cepacia* (*B. cepacia*).^1, 3, 4^ In spite of increased survival to date, most current CF treatments are symptomatic and hence target lung inflammation and infection rather than the primary CF defect, namely the loss of CFTR function. New anti-inflammatory drugs and antibiotics are in clinical trials in CF patients.^5, 6^

Bacterial infections and chronic lung inflammation have a pivotal role in CF lung disease and deeply influence each other, as microbial invasion leads to an uncontrolled inflammatory host–response that in turn favors unresolved bacterial infections and colonization.^3, 4, 6^ Whether the lack of CFTR channel activity directly drives inflammation is still debated. Emerging evidence indicates that CFTR does not only act as a pure ion channel, but also as a hub protein that orchestrates the proteostasis network (PN) and influence multiple cellular functions.^7, 8^ The dysfunction of this hub in CF deranges major mechanisms of cell physiology, both in primary epithelial cells biopsied from F508del-CFTR homozygous patients and in mice bearing an equivalent mutation in the mouse CFTR gene (CF mice). Autophagy, a major mechanism of cytoplasmic protein turnover, is defective in CF airways, owing to tissue transglutaminase (TG2)-mediated depletion of the essential autophagy-related protein Beclin 1 (BECN1).^8, 9, 10, 11, 12^ This leads to the accumulation of the autophagic substrate SQSTM1/p62,^8, 9, 10^ which sequestrates misfolded ubiquitinated F508del-CFTR and major anti-inflammatory proteins, such as PPAR*γ* and IK-B*α*, in intracellular aggresomes, thus sustaining a pro-inflammatory cellular environment.^8, 9, 10, 11, 12^ Notably, re-establishing a proper autophagy flux by means of genetic interventions (enforced expression of BECN1 or direct depletion of SQSTM1/p62) or the inhibition of TG2 by the proteostasis regulator cysteamine, controls lung inflammation in F508del-CFTR homozygous mice.^8, 9, 10^

Defective bacterial handling by macrophages is a feature of CF airways.^13, 14, 15, 16, 17, 18, 19^ CFTR is expressed by, and localizes at the plasma membrane of, human and mouse macrophages^6, 13, 14, 15, 16, 17, 18, 19^ and can also localize at the membranes of phagosomes and lysosomes.^13, 14, 15, 16^ Conflicting results have been reported on whether the defective bacterial killing observed in F508del-CFTR macrophages is the consequence of impaired acidification of lysosomes and/or unbalanced redox regulation due to defective channel function.^17, 18, 19, 20^ More recently, several authors reported that F508del-CFTR macrophages are also characterized by defective autophagy with reduced BECN1 levels and accumulation of SQSTM1/p62.^21, 22^

Emerging therapeutic approaches aim at controlling lung inflammation and ameliorating lung function through the repair of the primary CFTR defect.^6, 23, 24, 25^ These strategies take advantage from large-scale high-throughput screening programs that led to the identification of small molecules capable of either correcting the traffic of misfolded CFTR mutants (CFTR correctors) or improving channel function (CFTR potentiators) of plasma membrane-resident CFTR mutants. A CFTR potentiator (Ivacaftor, Kalydeco, Vertex Pharmaceutical) is available for a small fraction (5%) of CF patients bearing class III mutations (such as the G551D membrane-resident channel-dead CFTR mutant).^26^ Moreover, for the majority (>70%) of patients bearing the most common CFTR mutation, F508del-CFTR, the FDA approved the combination of the CFTR potentiator Ivacaftor with the corrector Lumacaftor (Orkambi), although this therapy is only marginally effective.^27, 28, 29, 30, 31^

Small molecules that target the deranged PN in CF (proteostasis regulators) can represent an alternative strategy to circumvent CFTR defect.^7, 8, 32, 33, 34^ We reported that a combination of two proteostasis regulators, cysteamine, an FDA-approved drug for the treatment of patients affected by cystinosis, and the over-the-counter nutraceutical epigallocatechin-gallate (EGCG) is effective in rescuing a functional F508del-CFTR to the cell surface and in stabilizing the rescued CFTR mutant at the plasma membrane of epithelial cells. Such a combinatory treatment succeeds in increasing survival and reducing lung inflammation *in vivo* in F508del homozygous mice.^8, 9, 10, 33, 34^ The effects of cysteamine are mediated by its ability to restore BECN1 expression and autophagy,^8, 9, 10^ whereas EGCG inhibits the master kinase CK2, responsible for CFTR fragmentation and degradation.^33, 35^ These pre-clinical results paved the way for 2 clinical trials in CF patients bearing F508del-CFTR, in whom the combination treatment restored autophagy, decreased sweat chloride concentrations and increased chloride efflux in brushed nasal cells.^33, 34^ Moreover, the treatment significantly reduced the levels of inflammatory cytokines in patients' sputum coupled with an amelioration of lung function.^33, 34^ Cysteamine is a known mucolytic agent and reduces the viscoelasticity of CF sputum.^36, 37^ It is also endowed with bactericidal effects against *P. aeruginosa* and other emerging pathogens and may disrupt established biofilms or prevent their formation in CF patients.^36, 37^ This may synergize with antibiotic treatments and help avoiding bacterial resistance.^33, 34^

Here, we addressed the question as to whether cysteamine might have a direct effect on the microbiocidal activity of macrophages, at least part of which depends on an intact autophagic machinery.^38, 39, 40, 41, 42, 43^ We show that cysteamine can restore the impaired ability of CFTR defective macrophages in killing *P. aeruginosa* through restoring BECN1 expression and autophagy.

## Results

### Cysteamine reverts defective killing of *P. aeruginosa* by BMDMs from CF mice

**Figure 1:**
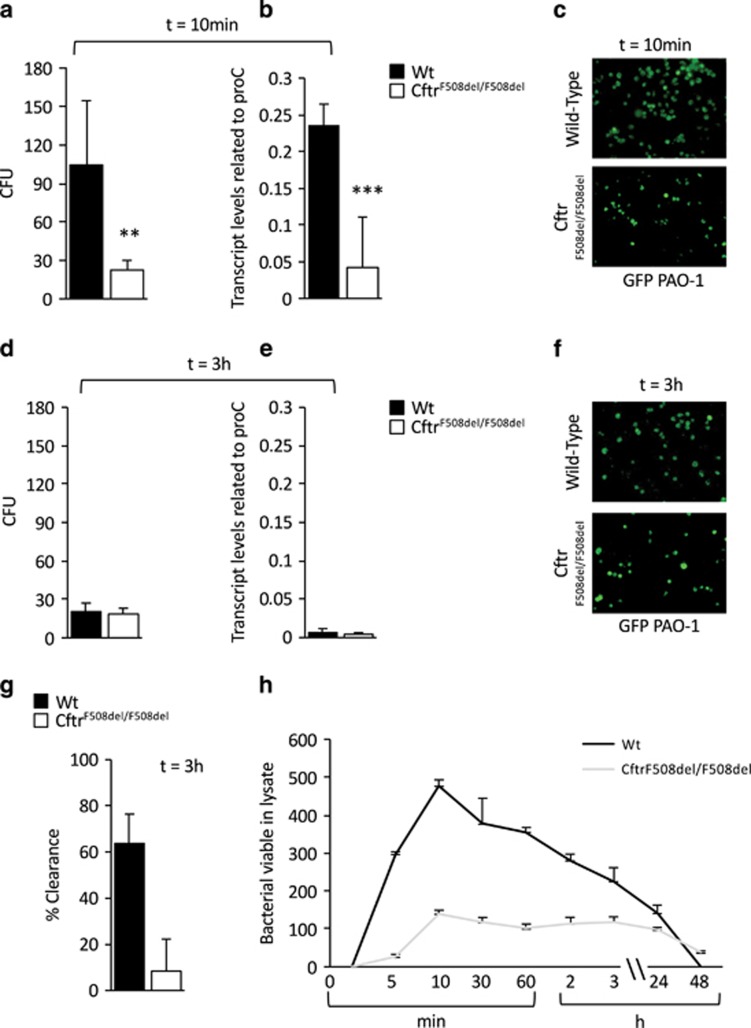
Internalization and clearance of *P. aeruginosa*-O1 (PAO1) in bone marrow-derived macrophages (BMDMs) from Wild-type (WT) and *Cftr*^*F508del/F508del*^ mice. (**a**–**g**) WT or *Cftr*^*F508del/F508del*^ BMDMs infected with PAO1 for 10 min followed by 10 min (**a**–**c**) or 3 h (**d**–**f**) of culture in the presence of gentamicin. (**a**, **d**) Number of colony-forming units (CFU) of PAO1 in WT (*n*=10) and *Cftr*^*F508del/F508del*^ cells (*n*=13) BMDMs (**P<0.01, Student's *t*-test) and (**b**, **e**) q-PCR transcript level of total PAO1 DNA in WT (*n*=3) and *Cftr*^*F508del/F508del*^ (*n*=3) BMDMs related to proC housekeeping gene (***P<0.001, Student's *t*-test). (**c**, **f**) Immunofluorescence detection of viable intracellular bacteria. (**g**) Clearance of bacteria expressed as percentage of living bacteria after 3 h of culture respect to the total amount of internalized bacteria in WT and *Cftr*^*F508del/F508del*^ BMDMs (**P<0.01, Student's *t*-test). (**h**) WT or *Cftr*^*F508del/F508del*^ BMDMs infected with PAO1-GFP at different time points. Enumeration of intracellular PAO1-GFP by immufluorescence in WT and *Cftr*^*F508del/F508del*^ BMDMs. Means±S.D. of three independent measurements for each group of experiments. Asterisks indicate significant differences

To determine whether cysteamine may improve the killing of *P. aeruginosa* upon acute infection, bone marrow-derived macrophages (BMDMs) were isolated from the femurs of 6–12 week-old CFTR homozygous mice in the *FVB/129* outbred background (below referred to as *Cftr*^*F508del/F508del*^), or their wild-type (WT) littermates.^21, 22^ Then the cells were inoculated with the *P. aeruginosa* strain PAO1 at a multiplicity of infection (MOI) of 50 bacteria/macrophage for 10 min, washed and treated for 10 min with 100 *μ*g/ml gentamicin to remove any extracellular bacteria and finally lysed to determine the clonogenic potential of surviving intracellular bacteria. To monitor the clearance of internalized bacteria, BMDMs were infected and treated as described, extensively washed and then kept in culture for 30 min, 1 h, 2 h, 3 h, and up to 48 h, as reported.^21, 22^ Colony-forming units (CFU) and bacterial DNA, together with the enumeration of the residual intracellular fluorescent-labeled *P. aeruginosa* were determined. To measure the effective intracellular *P. aeruginosa* clearance by BMDMs, we calculated the percentage of living bacteria after 3 h of culture with respect to the total amount of internalized bacteria. We observed that the internalization of *P. aeruginosa* was significantly lower in *Cftr*^*F508del/F508del*^ BMDMs than in WT controls (CFU, 22.5±7.3 *versus* 104.50±50, *P*<0.01 and bacterial DNA, 0.042±0.068 *versus* 0.23±0.028, *P*<0.001, respectively) (Figures 1a–c). Next, we measured the number of CFU, the amount of bacterial DNA and the number of the residual GFP-conjugated intracellular bacteria 3 h post-infection. Although the number of live bacteria was not significantly different in BMDMs isolated from *Cftr*^*F508del/F508del*^ mice (CFU, 18.6±4.5, bacterial DNA, 0.0042±0.0021) *versus* WT (CFU, 20.5±6.7, bacterial DNA, 0.0064±0.0052) mice (*P*>0.5) (Figures 1d–f), the effective clearance of internalized bacteria after 3 h of culture was greatly reduced in BMDMs from *Cftr*^*F508del/F508del*^ (8.3±13.9%) respect to WT littermates (63.7±12.7%, *P*<0.01) (Figure 1g). Moreover, viable bacteria were still detected as late as after 48 h following infection (Figure 1h). Thus, BMDMs from *Cftr*^*F508del/F508del*^ mice exhibit both defective internalization and impaired clearance of *P. aeruginosa*.

**Figure 2:**
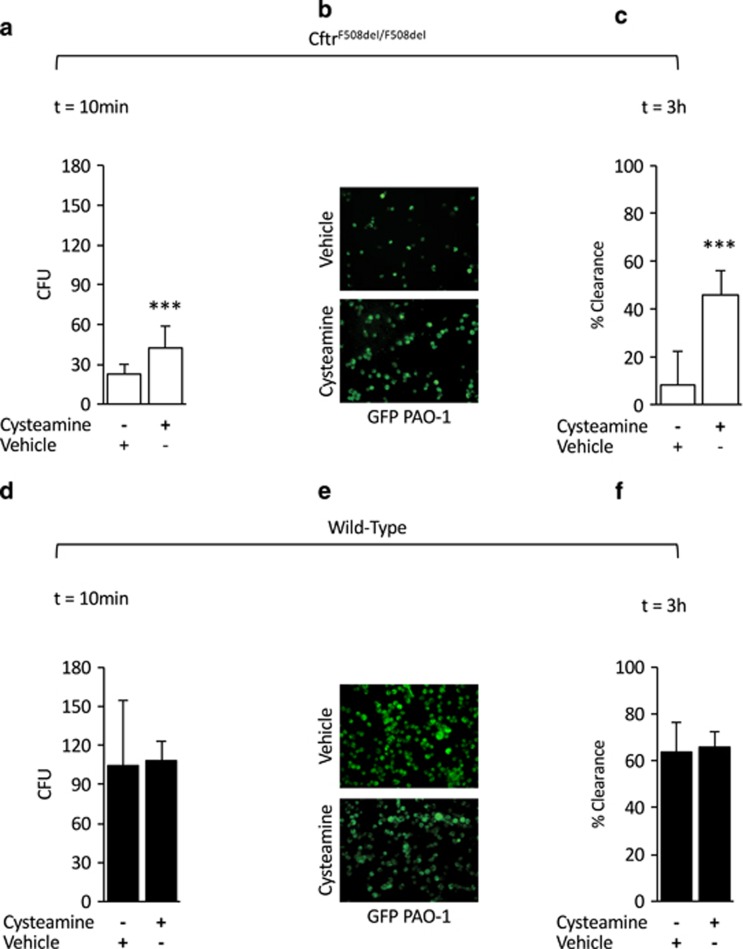
Effects of cysteamine on internalization and clearance of *P. aeruginosa*-O1 (PAO1) in bone marrow-derived macrophages (BMDMs) from wild-type (WT) and *Cftr*^*F508del/F508del*^ mice. BMDMs from *Cftr*^*F508del/F508del*^ (**a**–**c**) or WT (**d**–**f**) mice treated with 250 *μ*M cysteamine (*n*=10) or vehicle (*n*=10) for 10 min before infection followed by 10 min (**a**–**b**, **d**–**e**) or 3 h (**c**, **f**) of gentamicin culture. *P. aeruginosa* internalization expressed as number of CFUs of PAO1 (**a**, **d**) or enumerated by immunofluosescence (**b**, **e**) after 10 min of gentamicin culture (***P<0.001 *versus* vehicle; Student's *t*-test); (**c**, **f**) clearance of *P. aeruginosa* expressed as percentage of living bacteria after 3 h of culture respect to the total amount of internalized bacteria (***P<0.001 *versus* treatment with vehicle; Student's *t*-test). Means±S.D. of three independent measurements for each group of experiments. Asterisks indicate significant differences

To investigate whether cysteamine may modulate these events in BMDMs isolated from either WT and *Cftr*^*F508del/F508del*^ mice, the cells were pre-treated for 18 h with 250 *μ*M cysteamine or vehicle. Cysteamine significantly increased the internalization of *P. aeruginosa* (CFU, 42.3±16.3; *P*<0.001 *versus* vehicle-treated BMDMs) (Figures 2a and b) and improved the clearance of internalized *P. aeruginosa* in *Cftr*^*F508del/F508del*^ BMDMs up to 45.5±10.5% (*P*<0.001 as compared with vehicle-treated mice) (Figure 2c). No effects of cysteamine were observed in WT CFTR mice (Figures 2d–f).

### Cysteamine reverts defective killing of *P. aeruginosa* by restoring BECN1 expression and autophagy

**Figure 3:**
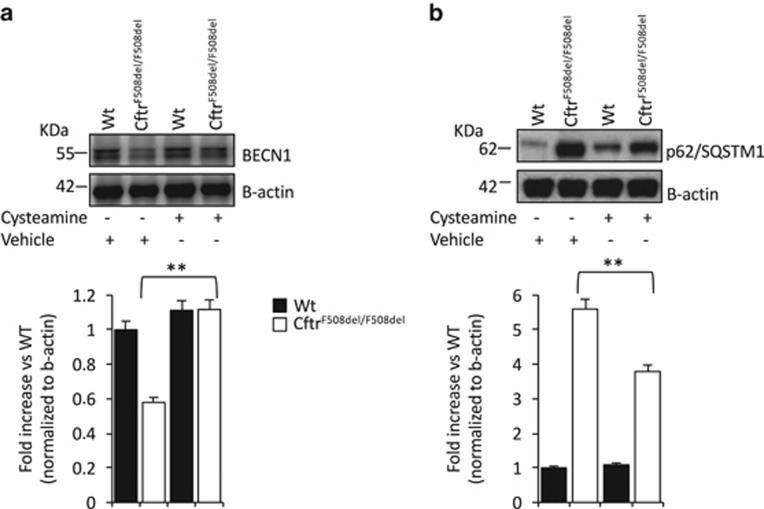
Effects of cysteamine on autophagy markers in bone marrow-derived macrophages (BMDMs). Wild-type (WT) or *Cftr*^*F508del/F508del*^ BMDMs treated for 18 h with cysteamine or vehicle. Representative immunoblots with anti-BECN1 (**a**) and anti-SQSTM1/p62 (**b**) (top) and densitometric measurements (bottom) in BMDMs from one mice per group. Mean±S.D. of three independent measurements (**P<0.01 *versus* vehicle-treated mice; ANOVA). Asterisks indicate significant differences

We previously reported that cysteamine restores autophagy in *Cftr*^*F508del/F508del*^ mice by preventing tissue TG2-mediated targeting of the master autophagy regulator BECN1 and its sequestration in intracellular aggregates.^9, 10, 11^ Previous studies reported that the colocalization of BECN1 and *B. cepacia* increases in F508del-CFTR macrophages upon autophagy induction.^21^ Accordingly, we found that cysteamine treatment of F508del-CFTR BMDMs increased BECN1 protein levels and reduced SQSTM1/p62 accumulation (Figures 3a and b).

**Figure 4:**
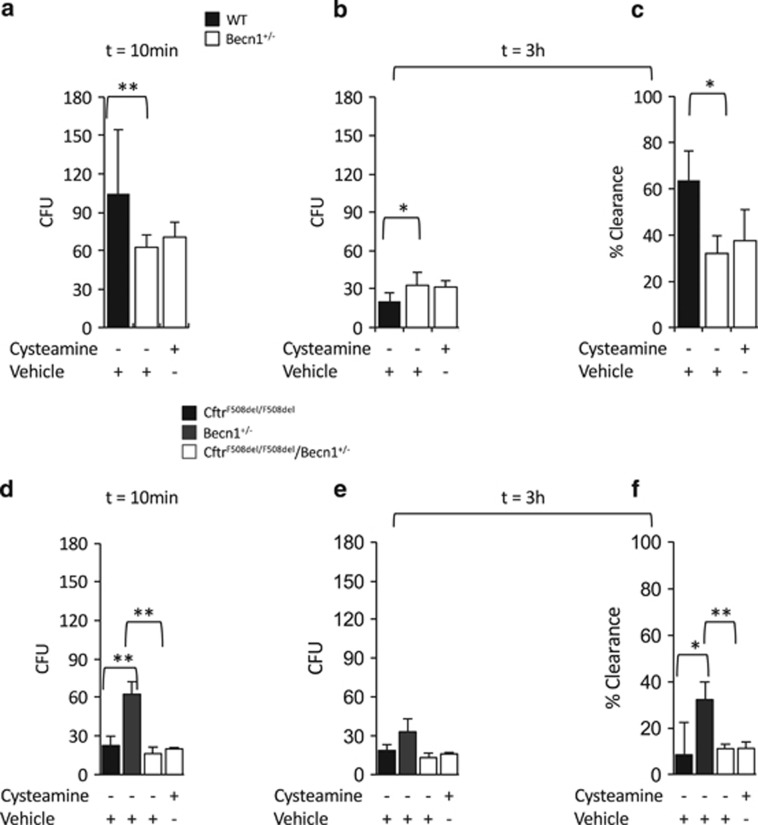
Cysteamine does not restore defective killing of *P. aeruginosa* by bone marrow-derived macrophages (BMDMs) from *Cftr*^*F508del/F508del*^*/Becn1*^**+/−**^ mice. BMDMs from wild-type (WT) (*n*=10) (**a**–**c**), Becn1 haplo-insufficient (*Becn1*^**+/−**^) (*n*=3) (**a**–**c**), *Cftr*^*F508del/F508del*^ (*n*=10) and *Cftr*^*F508del/F508del*^*/Becn1*^**+/−**^ (*n*=3) (**d**–**f**), mice infected with *P. aeruginosa*-O1 (PAO1) for 10 min followed by 10 min (**a**, **d**), or 3 h (**b**–**c** and **e**–**f**) of culture with gentamicin. Number of CFUs of PAO1 after 10 min (**a**, **d**) or 3 h (**b**, **e**); clearance of PA expressed as percentage of living bacteria after 3 h of culture respect to the total amount of internalized bacteria (**c**, **f**). Effects of the pre-treatment with cysteamine in BMDMs from *Becn1*^**+/−**^ (**a**–**c**) and *Cftr*^*F508del/F508del*^*/Becn1*^+/−^ (**d**–**f**) mice. Means±S.D. of three independent measurements for each group of experiments. *P<0.05, **P<0.01, Student's *t*-test

To provide the genetic proof for the involvement of BECN1 in bacterial killing of *P. aeruginosa*, BMDMs were isolated from *Becn*^+/−^ haplo-insufficient mice on a *C57BL/6 J* background and infected with *P. aeruginosa*. Macrophages from *Becn1*^+/−^ haplo-insufficient mice efficiently internalized *P. aeruginosa* (CFU, 62.6±9.7) as compared with WT (*P*>0.5). In contrast, they manifested defective bacterial clearance with a percentage of clearance of 32+7.6% (*P*<0.05) (Figures 4a–c). This suggests a critical role of BECN1 and autophagy in the clearance of the internalized bacteria but not in the earliest steps of *P. aeruginosa* uptake. Notably, cysteamine was not capable of increasing the intracellular clearance of *P. aeruginosa* (*P*>0.5) in BMDMs from *Becn1*^+/−^ upon infection (Figure 4c).

To unveil the role of BECN1 in a F508del-CFTR defective context, *Cftr*^F508del**/+**^ female mice were backcrossed with *Becn1*^+/−^ mice to obtain *Becn1* haplo-insufficient F508del heterozygous mice (abbreviated *Cftr*^F508del/+^/*Becn1*^+/−^) at the first generation. These *Cftr*^F508del/+^/*Becn1*^+/−^ animals were crossbred to obtain *Becn1* haplo-insufficient F508del homozygous mice (abbreviated *Cftr*^F508del/F508del^/*Becn1*^+/−^).^34^ In BMDMs from *Cftr*^F508del/F508del^/*Becn1*^+/−^ mice, both *P. aeruginosa* uptake (CFU, 16.3±5.1) and clearance (CFU, 13±3.6) were greatly reduced as compared with either WT or their *Becn1*^+/−^ and were similar to those in *Cftr*^*F508del/F508del*^ mice (Figures 4d–f). Notably, cysteamine showed negligible effects on either uptake and clearance of *P. aeruginosa* by *Cftr*^F508del/F508del^/*Becn1*^+/−^ BMDMs (*P*>0.5) (Figures 4d–f). These results indicate that cysteamine enhances bacterial clearance of *P. aeruginosa* through restoring BECN1 expression and autophagy.

### Cysteamine reverts defective killing of *P. aeruginosa* by rescuing a functional CFTR protein

**Figure 5:**
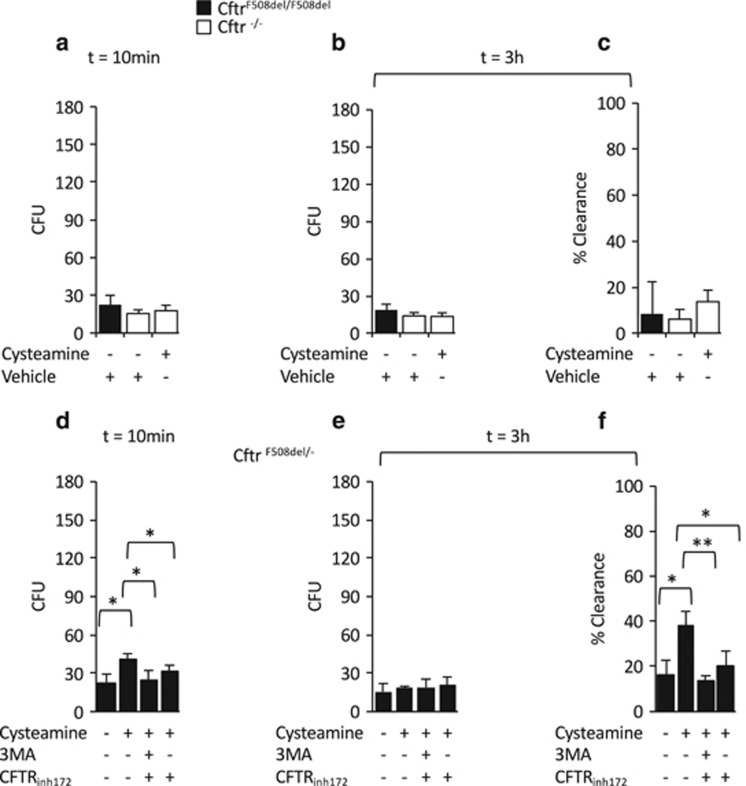
Cysteamine reverts defective killing of *P. aeruginosa* through rescuing CFTR. Bone marrow-derived macrophages (BMDMs) from *Cftr*^*F508del/F508del*^ (*n*=3) (**a**–**c**), *Cftr*^−/−^ (*n*=3) (**a**–**c**), and Cftr^F508del/−^ (*n*=3) (**d**–**f**), and infected with *P. aeruginosa*-O1 (PAO1) for 10 min followed by 10 min (**a**, **d**) or 3 h (**b**–**c** and **e**–**f**) of culture in the presence of gentamicin. (**a**–**b**, **d**–**e**) number of CFU of PAO1; (**c**, **f**) clearance of bacteria expressed as percentage of living bacteria after 3 h of culture respect to the total amount of internalized bacteria. BMDMs from *Cftr*^−/−^ (*n*=3) (**a**–**c**) and Cftr^F508del/−^ (**d**–**f**) mice treated with 250 *μ*M cysteamine or vehicle in the presence or absence of 3-methyl-adenine (3-MA) (3 mM) and/or CFTR_inh172_ (20 *μ*M) (**d**–**f**) and then infected with PAO1 for 10 min followed by 10 min (**a**, **d**) or 3 h (**b**–**c** and **e**–**f**) of gentamicin culture. *P<0.05, **P<0.01, Student's *t*-test. Means±S.D. of three independent measurements for each group of experiments. Asterisks indicate signifcant differences

Cysteamine is effective in either rescuing and stabilizing misfolded F508del-CFTR mutant at the PM.^9, 10^ Notably, cysteamine reduces lung inflammation *in vivo* through re-establishing CFTR function either in *Cftr*^F508del/F508del^ mice or in CF patients bearing class II CFTR mutations.^9, 10, 33, 34^ To determine whether the effects of cysteamine on bacterial killing are secondary to its ability to rescue CFTR function, BMDMs were isolated from mice in which the gene coding for CFTR has been knocked out by homologous recombination (i.e., *Cftr*^−/−^ mice). Both bacterial internalization (CFU, 15.5±2.88) and intracellular clearance of *P. aeruginosa* (6.08±4.18% of clearance) were similar to those observed in *Cftr*^F508del/F508del^ mice (*P*>0.5) (Figures 5a–c). However, the treatment with cysteamine failed to restore the uptake (CFU, 17.66±4.5) or clearance (CFU, 13.33±2.88) of *P. aeruginosa* by *Cftr*^−/−^ BMDMs (Figures 5a–c), contrasting with its significant beneficial effects on BMDMs from *Cftr*^F508del/F508del^ mice.

To provide the proof-of-concept that the beneficial effects of cysteamine on *P. aeruginosa* killing rely on the presence of a rescuable CFTR mutant, heterozygous *Cftr*^F508del**/+**^males were backcrossed with heterozygous *Cftr*^+/−^ females to obtain F508del/null CFTR heterozygous mice (abbreviated *Cftr*^F508del^/^−^). BMDMs were infected and treated with cysteamine in the presence or absence of the autophagy inhibitor 3-methyl-adenine (3-MA) before infection. BMDMs from *Cftr*^F508del^/^−^ mice behaved similarly to *Cftr*^F508del/F508del^ mice, in thus far that cysteamine was effective in restoring *P. aeruginosa* uptake (22.3±7.02 CFU in controls: and 41±4.35, after cysteamine treatment; *P*<0.05) or clearance (16.5±6.28 of clearance in controls and 38.02±6.45% of after cysteamine treatment, *P*<0.05), unless 3-MA was also added to the culture system (CFU, 25±7.2 and 14.05±1.85% of clearance) (Figures 5d–f). These results provide the genetic proof that cysteamine restores bacterial killing through rescuing a functional F508del-CFTR and hence re-establishing BECN1-dependent autophagic flux. Accordingly, the addition of the CFTR inhibitor 172 (CFTR_inh172_) to the system during cysteamine treatment, significantly reduced the beneficial effects of cysteamine on the internalization and clearance of *P. aeruginosa* in both *Cftr*^F508del/F508del^ and *Cftr*^F508del^/^−^ mice (Figures 5d–f).

### Cysteamine decreases the production of inflammatory cytokines induced by *P. aeruginosa* in BMDMs

**Figure 6:**
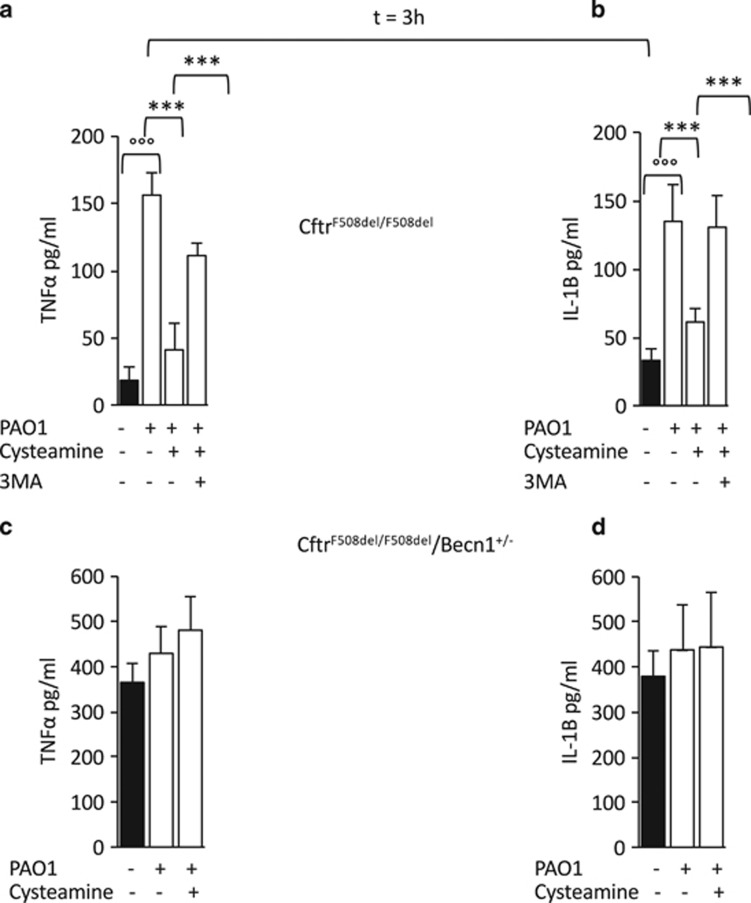
Cysteamine controls *P. aeruginosa*-induced increase of TNF*α* and IL-1*β* levels in bone marrow-derived macrophages (BMDMs) from *Cftr*^*F508del/F508del*^ mice. BMDMs from *Cftr*^*F508del/F508del*^ (*n*=3) (**a** and **b**) and *Cftr*^*F508del/F508del*^*/Becn1*^**+/−**^ (*n*=3) (**c** and **d**) mice treated with 250 *μ*M cysteamine in the presence or absence of 3-methyl-adenine (3-MA) (3 mM) (**a** and **b**) and then infected with *P. aeruginosa*-O1 (PAO1) for 10 min followed by culture with gentamicin up to 24 h. Quantitation of TNF*α* (**a**, **c**) and IL-1*β* (**b**, **d**) levels in culture supernatants by ELISA. ***P<0.001, °°°*P*<0.001, Student's *t*-test. Means±S.D. of three independent measurements for each group of experiments. Asterisks indicate signifcant differences

To explore the possibility that cysteamine may control the increased production of inflammatory cytokines by BMDMs from *Cftr*^F508del/F508del^ mice following *P. aeruginosa* infection, we measured the levels of IL-1*β* and TNF-*α* in culture supernatants from BMDMs collected after 24 h following infection. Pre-treatment with cysteamine reduced the levels of pro-inflammatory cytokines as compared to untreated control cells (Figures 6a and b). These beneficial effects of cysteamine were abrogated if 3-MA (Figures 6a and b) or wortmannin (Supplementary Figure 1) were added to the culture system. No effect of cysteamine on cytokine levels was observed in BMDMs from *Cftr*^F508del/F508del^/*Becn1*^+/−^ mice (Figures 6c and d). Hence, haploinsufficiency of *Becn1* curtails the anti-inflammatory effects of cysteamine on *Cftr*^F508del/F508del^ BMDMs.

## Discussion

Persistent infections by *P. aeruginosa* indicate dismal prognosis for CF patients.^44^ Although chronic bacterial adaptation to the CF lung environment can favor colonization and antibiotic resistance, intrinsic host–macrophage defects compromise the capacity of CF phagocytes to properly fight bacterial challenges,^13, 14, 15, 16, 17, 18, 19, 20^ thus favouring chronic infection. The mechanisms underlying defective bacterial killing in CF are not completely understood. Conflicting results have been reported on mouse and human CF macrophages, which were either monocyte-derived or lung-resident,^13, 14, 15, 16, 17, 18, 19, 20^ likely because the latter are conditioned by the pro-inflammatory and micro-anaerobiotic environment of CF airways. Bacterial killing is a complex mechanism resulting from a sequential cascade of events, which depend on bacterial strain, environmental conditions^13, 14, 15, 16, 17, 18, 19, 20^ together with the ability of the host phagocytes to properly internalize and clear bacteria.^13, 14, 15, 16, 17, 18, 19, 20^

CFTR is expressed by human and mouse macrophages.^18, 19^ However, whether and how defective CFTR has a direct role in bacterial killing is still debated. Conflicting results were reported on whether defective CFTR impairs the acidification of the lysosome or deranges the clearance of *P. aeruginosa* via redox-dependent processes.^17, 18, 19, 20^ More recently, impaired autophagy has been involved in the defective killing of *B. cepacia* by CF macrophages.^21, 22^ Autophagy is a general mechanism of survival that cells adopt to cope with stress through targeting cytosolic or recently internalized proteins to lysosomal degradation.^38, 39, 40, 41, 42, 43^ Defective autophagy was first discovered as a major consequence of defective CFTR in bronchial epithelial cells^9, 10, 45^ and subsequently described in CF macrophages.^21, 22^ Depletion of SQSTM1/p62 from F508del-CFTR macrophages *in vitro* improves autophagy, increases the colocalization of *B. cepacia* with the master autophagy protein BECN1 and reduces intracellular *B. cepacia* survival.^21, 22^ We have previously described that defective autophagy in CF is due to TG2-mediated sequestration of BECN1, thus reducing the BECN1-dependent generation of phosphatidyl-inositol-3-phosphate (*PI3P*) that is important for increasing autophagic flux.^9, 10, 45, 46^

Our results provide genetic proof for the role of CFTR and autophagy in orchestrating the sequential cascade of events following the acute infection of *P. aeruginosa* in BMDMs (which are non-conditioned phagocytes). We used a panoply of established and newly generated transgenic mice bearing different CFTR defects, such as *Cftr*^−/−^ mice (which lack any rescuable CFTR protein) or mice bearing F508del-CFTR in homozygosis (*Cftr*^F508del/F508del^ mice) or in combination with a null CFTR allele (*Cftr*^F508del^/^−^ mice). Although murine models of CF do not recapitulate the clinical manifestations of CF lung disease, CF mice manifest an inflammatory lung phenotype coupled to hyper-responsiveness to bacteria or microbial products and are widely used to test candidate drugs and their mechanism of action.^47^ In addition, we used autophagy-defective *Becn1*^**+/−**^ mice as well as *Cftr*^F508del/F508del^ mice in a background of BECN1 haploinsufficiency (*Cftr*^F508del/F508del^/*Becn1*^**+/−**^ mice). First, we analyzed the early steps following acute infection. As soon as 10 min following infection, CFTR competent BMDMs are able to internalize bacteria, whereas BMDMs isolated from either *Cftr*^−/−^ or *Cftr*^F508del/F508del^ or *Cftr*^F508del^/^−^ mice exhibited a major internalization defect. Notably, the CFU enumerated after 3 h of incubation following infection, were similar in WT and CF mice. However, the percentage of clearance of internalized bacteria was impaired in BMDMs from CF mice with distinct genotypes (*Cftr*^−/−^, *Cftr*^F508del^/^−^*, Cftr*^F508del/F508del^), indicating that CFTR defective macrophages are unable to properly clear internalized bacteria. The defect of *P. aeruginosa* internalization was not secondary to defective BECN1 function or reduced autophagy as it was not observed in *Becn1*^**+/−**^ mice that, however, manifested reduced intracellular clearance of internalized bacteria to approximately a half of the values of their autophagy-competent littermates. Accordingly, when *Cftr*^F508del/F508del^ were backcrossed in a BECN1 haplo-insufficient background (*Cftr*^F508del/F508del^/*Becn1*^**+/−**^ mice), the response of BMDMs to the acute *P. aeruginosa* infection was similar to that observed in *Cftr*^F508del/F508del^ mice.

Cysteamine was effective in restoring BMDMs dysfunction from either *Cftr*^F508del/F508del^ and *Cftr*^F508del^/^−^, but not *Cftr*^−/−^ transgenic mice. This supports the hypothesis that cysteamine exerts its beneficial effects on CF through restoring a functional F508del-CFTR, in agreement with our previous data in transgenic mice and in CF patients bearing class II CFTR mutations. In contrast, cysteamine failed to have any beneficial effect of *P. aeruginosa* uptake or clearance by BMDMs when CFTR protein was not rescuable, namely in *Cftr*^−/−^ BMDMs. Moreover, cysteamine was not effective on *Cftr*^F508del/F508del^/*Becn1*^**+/−**^ BMDMs as well as on BMDMs from *Cftr*^F508del/F508del^ that were simultaneously treated with the autophagy inhibitor 3-MA. Altogether these results indicate that cysteamine can rescue a WT like phenotype in CF macrophages through restoring autophagy, exactly as it does in CF epithelial cells.

Unresolved inflammation is a major feature of CF lungs. Defective CFTR generates a dysregulation of the innate immune response at the mucosal sites facing airway and gut lumen.^1, 3, 4^ This leads to an unbalanced response to either cell-autonomous or luminal stressors, as it is the case of bacterial infections in airways. Such a constitutive, yet inefficient, activation of the innate immune response increases the susceptibility of CF patients to manifest recurrent and unresolved bacterial infections that further increase lung inflammation.

Therefore, defective *P. aeruginosa* internalization coupled to excessive and unbalanced activation of mucosal immune response is a puzzling feature of CF airways. The mechanisms responsible for *P. aeruginosa* internalization in different cell types and tissues are complex and not completely understood. CFTR has a key role in the formation of an internalization platform that requires cytoskeleton integrity and the cooperation of the essential scaffold protein Caveolin 1 (CAV-1).^15^ CF macrophages fail to enhance CAV-1 expression in response to LPS^15^ and also show defective compartimentalization of heme-oxygenase 1 (HO-1),^48^ a regulator of redox balance induced by stress conditions including infections, to the cell surface where the CAV-1/HO-1 complex acts as negative regulator of TLR-4 signaling.^15, 48, 49^ Thus, defective CFTR compromises major mechanisms of bacterial internalization by either mouse and human macrophages, while sustaining an inappropriate inflammatory response. These puzzling features of CF macrophages may explain how even low bacterial challenges may result in an exaggerated inflammatory response due to defective elimination of internalized bacteria coupled to sustained TLR signaling.

In conclusion, our study provides genetic proof on how the interplay between defective CFTR and impaired autophagy compromises the ability of CF macrophages to fight *P. aeruginosa* infection. Our results offer a new perspective for the treatment of *P. aeruginosa* infection in CF patients, as they provide the proof-of-concept for the beneficial effect of cysteamine in reverting macrophage dysfunction in CF. Recurrent and chronic bacterial infections represent a major problem in the treatment of CF patients and entail the need of long-term and expensive antibiotic treatments with the risk of adverse reactions and antibiotic resistance. Cysteamine is a repurposed drug, with a known safety profile. Combining the effects of cysteamine on CFTR rescue and lung inflammation with the ability to improve bacterial clearance by macrophages, as we describe in this study, might result in long-term clinical benefit to CF patients bearing the F508del-CFTR mutation. At this point, we surmise that this speculation warrants further clinical evaluation by investigating primary macrophages from cysteamine-treated CF patients.

## Materials and methods

### Mice and treatments

CF mice homozygous for the F508del-CFTR in the FVB/129 outbred background (Cftrtm^1EUR^, F508del, FVB/129, abbreviated *Cftr*^*F508del/F508del*^) were obtained from Bob Scholte, Erasmus Medical Center Rotterdam, The Netherlands, CF coordinated action program EU FP6 LSHMCT-2005-018932.^50^ Transgenic KO Cftr mice (B6.129P2-KOCftrtm^1UNC^, abbreviated *Cftr*^−/−^), were purchased from The Jackson Laboratory (Bar Harbor, ME, USA). The heterozygous *Cftr*^*F508del/+*^ males were backcrossed with the heterozygous *Cftr*^+/−^ females to obtain F508del/null CFTR heterozygous mice (abbreviated *Cftr*^*F508del/−*)^.^34^ *Cftr*^*F508del/+*^ female mice were backcrossed to the C57BL/6J background Becn1^+/–^ male mice (generous gift from Beth Levine, Center for Autophagy Research, Department of Internal Medicine, UT Southwestern Medical Center, Dallas, USA and Francesco Cecconi, University of Tor Vergata, Rome, Italy) to obtain at the first generation Becn1 haplo-insufficient F508del heterozygous mice (abbreviated *Cftr*^*F508del/+*^*/Becn*^*1+/−*^). These mice *Cftr*^*F508del/+*^*/Becn1*^+/−^ were crossbred to obtain Becn1 haplo-insufficient F508del homozygous mice (abbreviated *Cftr*^*F508del/F508del*^*/Becn*^*1+/−*^).^34^ The newly generated *Cftr* ^*F508del/−*^ and the *Cftr*^*F508del/F508del*^*/Becn1*^*+/–*^ were housed at the San Raffaele Scientific Institute SOPF animal house (Milan, Italy). These mice were provided with a special food, consisting of an equal mixture of SRM-A (Arie Blok, Woerden, The Netherlands) and Teklad 2019 (Harlan Laboratories, San Pietro al Natisone, Udine, Italy) and water acidified to pH 2.0 with HCl and containing 60 g/l PEG 3350, 1.46 g/l NaCl, 0.745 g/l KCl, 1.68 g/l NaHCO, and 5.68 g/l Na2SO4. Newborn mice were genotyped by cutting a small piece of tail 12 days after birth. DNA was extracted by digesting tails with Direct PCR Lysis Reagent (Viagen, CA, USA) and 1 mg/ml Proteinase K overnight at 56 °C. For Cftr^F508del/−^ two PCR reactions were performed. For the *Cftr*^*F508del/F508del*^ mutation, thermocycling consisted of an initial polymerase activation step at 95 °C for 5 min, amplification was performed with 30 cycles of 95 °C for 1 min, 52 °C for 1 min and 72 °C for 1 min with a final extension at 72 °C for 2 min; for the *Cftr*^−/−^ mutation thermocycling consisted of an initial polymerase activation step at 94 °C for 3 min, amplification was performed with 30 cycles of 94 °C for 30 s, 57 °C for 30 s and 72 °C for 30 s with a final extension at 72 °C for 10 min. For *Cftr*^*F508del/F508del*^*/Becn1*^*+/–*^, after analyzing the Cftr^F508del/F508del^ mutation, the Becn1^+/–^ thermocycling consisted of an initial polymerase activation step at 95 °C for 4 min, amplification was performed with 30 cycles of 95 °C for 1 min, 62 °C for 1 min and 72 °C for 1.30 min with a final extension at 72 °C for 10 min. Mice were anesthetized with Avertine (tribromoethanol, 250 mg/kg, Sigma Aldrich, Milan, Italy, T48402) and then killed and bone marrow was collected. All the procedures in mice were approved by the local Ethics Committee for Animal Welfare (IACUC No. 713) and were carried out in strict respect of European and National regulations.

### Bacteria

*P. aeruginosa* strain PAO1 or PAO1-GFP (kindly provided by EM Bruscia, Yale University School of Medicine) was used for these studies. Bacteria were kept frozen at −80 °C in 10% glycerol. Bacteria were thawed and grown in broth LB (Sodium Chloride, Sigma Aldrich, bacto tryptone, BD yeast extract, BD) overnight. The concentration of bacteria was determined by spectrophotometric reading at 600 nm, as reported.^51^

### Isolation and culture of BMDMs

BMDMs were isolated from the femurs of 6- to 12-wk-old mice and cultured as previously described.^21, 22^ After overnight culture, the non-adherent cells were differentiated for 7 days in 20 ng/ml recombinant M-CSF (ConnStem Inc., CT, USA). After 7 days, cells were detached and characterized by flow cytometry (F4-80+/MAC-1+ population). We obtained 1–3 × 10^7^ mature macrophages for each mice.

### BMDMs treatments

Macrophages were treated for 18 h with medium or cysteamine, (250 *μ*M, Sigma Aldrich, M9768), with or without the autophagy inhibitor 3-MA (3 mM, Sigma Aldrich, M9281) or wortmannin (0.5 *μ*M Sigma Aldrich, W1628) or the CFTR inhibitor (CFTR_inh-172_) (20 *μ*M, Sigma Aldrich, C2992) and then infected in the presence or absence of drugs. The cells were infected, with PAO1 or PAO-1GFP at a MOI of 50 bacteria/macrophage for 10 min and then were washed and treated with gentamicin (100 *μ*g/ml, Sigma Aldrich, G1397) for 10 min and then lysed or extensively washed and kept in culture up to 3 h or 24 h. The cells were lysed with a solution 0.2% triton x-100 (Biorad, Milan, Italy 1610407) and then plated in agar petridish in presence of carbenicillin (100 *μ*g/ml, Sigma Aldrich, C1613) overnight at 37 °C. CFU were enumerated as desctibed.^21, 22^ In addition, the enumeration of the residual intracellular fluorescent-labelled PA, was determined as reported.^21, 22^ The effective intracellular *P. aeruginosa* clearance by BMDMs, was determined by calculating the percentage of living bacteria after 3 h of culture with respect to the total amount of internalized bacteria.

### Immunofluorescence

The microscopy samples were analyzed with an Axivert 200LSM5 Meta microscope (Zeiss, NY, USA). Two million BMDMs were cultured on 12 mm glass cover slips in 24-well tissue culture plates and infected synchronously with PAO1-GFP at an MOI of 50 bacteria/macrophage. The BMDMs were fixed with PFA 4%. All experiments were performed in at least triplicate.

### Immunoblot analysis

Western blot analysis was performed as previously described^9, 10^ with antibodies against the following proteins: BECN1, 1:1000, (Abcam, Cambridge, UK Ab55878), *β*-actin 1:1000, (Cell Signaling Inc., Danvers, MA, USA, 4970), SQSTM1/p62 1:1000, (Sigma Aldrich, MABN130). The densitometric analysis was performed by Image J software and each data point was expressed as the mean±S.D. of independent experiments.

### Real-time PCR

Total RNA was extracted with the RNeasy Mini Kit (Qiagen, Milan, Italy, 74104). The mRNA was reverse transcribed with a SuperScriptTM III First Strand Synthesis System (Promega, Milan, Italy, A5001). Quantitative RT-PCR was performed with an iCycler iQ Multicolor Real-Time PCR Detector (BioRad) with iQ TM SYBR Green supermix (Five Prime, 2900217). The relative amounts of mRNA were calculated by using the comparative Ct method. Real-time RT-PCR analyses were executed for evaluating the efficiency of expression. Thermocycling consisted of an initial polymerase activation step at 98 °C for 5 min, and amplification was performed with 35 cycles of 95 °C for 15 s, 68 °C for 10 s, and 72 °C for 20 s with data acquisition at this stage and the reaction finished by the built in melt curve. Expression levels of genes were normalized to the housekeeping gene proC in the same sample.

The sequence of PAO1 was: lasB forward 5′-ATGAACGAGGCGTTCTCCG-3′and reverse 5′-GTTGTACACGCCGCTGGAGT-3′.

Housekeeping gene proC forward 5′-CAGGCCGGGCAGTTGCTGTC-3′ and reverse 5′-GGTCAGGCGCGAGGCTGTCT-3′.

### Cytokine assessment

Levels of IL-1*β* and TNF-*α* in culture supernatants from BMDMs collected 24 h following infection were assessed by means of standard ELISA kits (R&D Systems, Minneapolis, MN, USA), according to the manufacturer's instructions, as reported.^33, 34^ Samples were read in triplicate at 450 nm in Microplate Reader (BioRad) using Microplate Manager 5.2.1 software. Values were normalized to protein concentration evaluated by Bradford analysis.

### Statistical analysis

Data are reported as arithmetic mean±S.D. Data distribution was analyzed for normality and comparisons between groups for statistical difference were done using Student's two tailed *t*-test or the one-way ANOVA, as indicated. Significant differences are indicated in the figures. All experiments were performed at least three independent times with similar results. Data were analyzed using SPSS 20 software. Statistical significance was defined as *P*-value of <0.05.
