# Supplementary material for: Cysteamine re-establishes the clearance of Pseudomonas aeruginosa by macrophages bearing the cystic fibrosis-relevant F508del-CFTR mutation
Source: Cell Death Dis. 2017 Jan 12;8(1):e2544–. doi: 10.1038/cddis.2016.476 (PMC5386380; doi:10.1038/cddis.2016.476)
Supplement: Supplementary Material [file cddis2016476x1.pdf]

Cysteamine re-establishes the clearance of *Pseudomonas aeruginosa* by macrophages bearing the cystic fibrosis-relevant F508del-CFTR mutation

Eleonora Ferrari , Romina Monzani , Valeria Rachela Villella , Speranza Esposito , Francesca Saluzzo , Federica Rossin , Manuela D'Eletto , Antonella Tosco , Fabiola De Gregorio , Valentina Izzo , Maria Chiara Maiuri , Guido Kroemer , Valeria Raia and Luigi Maiuri

Supplementary Figures S1
Supplementary Figure Legend
Supplementary Material and Methods

Supplementary Figure 1

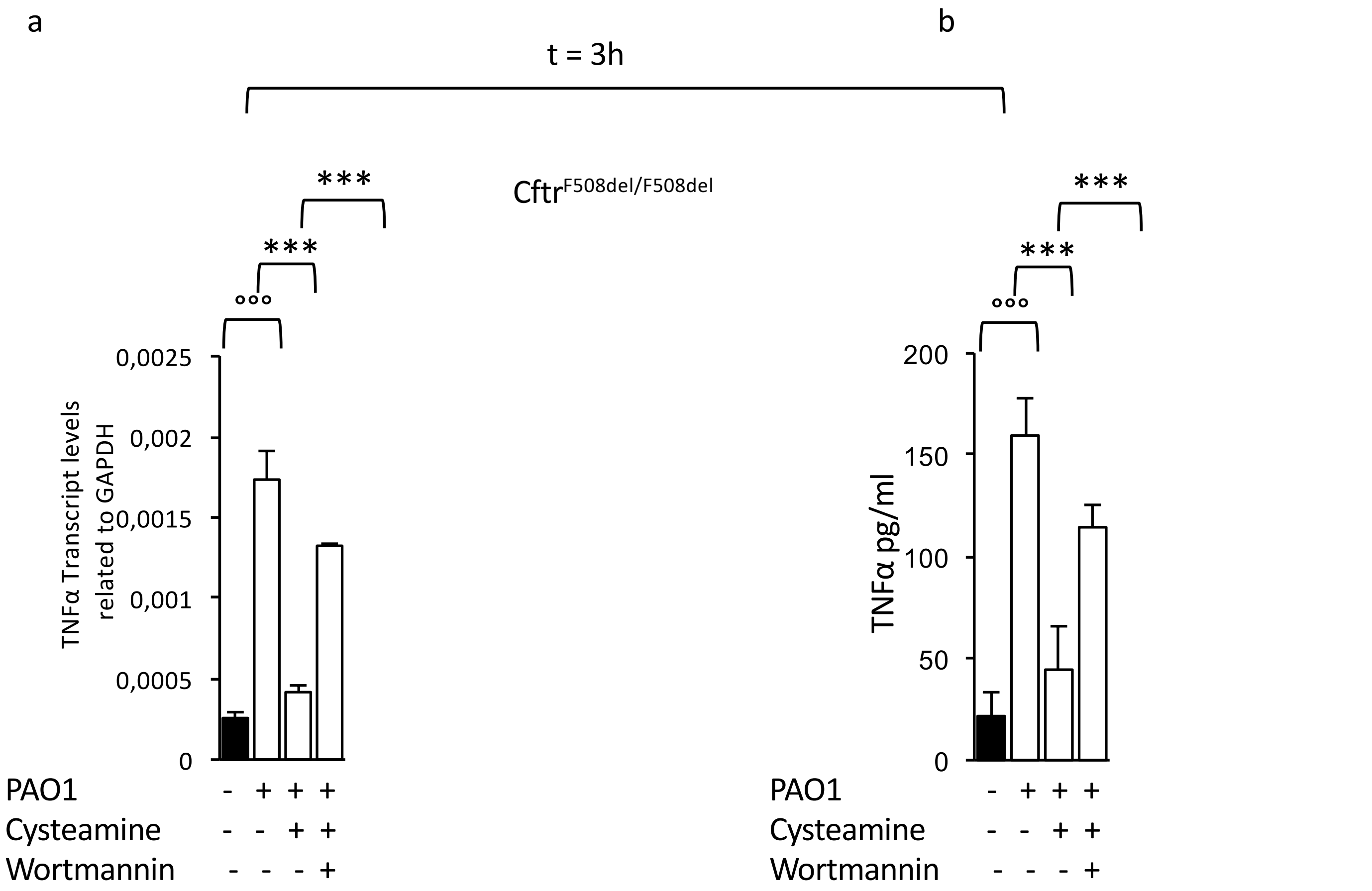

Supplementary Fig.1: Effects of wortmannin on TNFα production following PA infection. Cftr$^{F508del/F508del}$ (n=3) BMDMs were treated with cysteamine 250uM with or without wortmannin (0.5 µM) and then infected with PAO1-GFP for 10 min followed by 3h of gentamycin culture. a) TNFα transcript levels assessed by q-PCR and b) TNFα levels measured by ELISA, after 3h following gentamycin culture. °°°p<0.001 vs PAO1, ***p<0.001 vs Cysteamine; Student t test). Data are representative of 3 independent experiments and presented as the means ± SD. Asterisks indicate signifcant differences

Supplementary Material and Methods

Real Time PCR

Total RNA was extracted with the RNeasy Mini Kit (Qiagen, 74104). The mRNA was reverse transcribed with a SuperScriptTM III First Strand Synthesis System (Promega, A5001). Quantitative RT-PCR was performed with an iCycler iQ Multicolor Real-Time PCR Detector (Bio-Rad, Milan, Italy) with iQ TM SYBR Green supermix (Five Prime, 2900217). The relative amounts of mRNA were calculated by using the comparative Ct method. Real-time RT-PCR analyses were executed for evaluating the efficiency of expression. Thermocycling consisted of an initial polymerase activation step at 98°C for 5 min, and amplification was performed with 35 cycles of 95°C for 15 sec, 68°C for 10 sec and 72°C for 20 sec with data acquisition at this stage and the reaction finished by the built in melt curve. Expression levels of genes were normalized to the housekeeping gene proC in the same sample.

The sequence of TNFα was: forward 5′-CCACCACGCT CTTCTGTCTA-3′ and reverse 5′-AGGGTCTGGG CCATAGAACT-3′

The sequence of GAPDH was: forward 5'-GTGATGCTGGGTG-3‘ and reverse 5'-CAGTCTTCTGAG-3'
